# Long term administration of loquat leaves and their major component, ursolic acid, attenuated endogenous amyloid-β burden and memory impairment

**DOI:** 10.1038/s41598-023-44098-3

**Published:** 2023-10-05

**Authors:** Kensuke Iwasa, Sosuke Yagishita, Nan Yagishita-Kyo, Anzu Yamagishi, Shinji Yamamoto, Kota Yamashina, Chikara Haruta, Masashi Asai, Kei Maruyama, Kuniyoshi Shimizu, Keisuke Yoshikawa

**Affiliations:** 1 Department of Pharmacology, Faculty of Medicine, Saitama Medical University, 38 Moro-Hongo, Moroyama-Machi, Iruma-Gun, Saitama, 350-0495 Japan; 2 Laboratory of Kampo Pharmacology, Faculty of Pharmaceutical Sciences, Yokohama University of Pharmacy, Kanagawa, 245-0066 Japan; 3 Laboratory of Systematic Forest and Forest Products Sciences, Division of Sustainable Bioresources Science, Department of Agro-Environmental Sciences, Faculty of Agriculture, Kyushu University, Fukuoka, 819-0395 Japan

**Keywords:** Neuroscience, Medical research, Neurology

## Abstract

Loquat (*Eriobotrya japonica*) leaves contain many bioactive components such as ursolic acid (UA) and amygdalin. We investigated the effects of loquat leaf powder and methanol extract in human neuroglioma H4 cells stably expressing the Swedish-type APP695 (APP_NL_-H4 cells) and C57BL/6 J mice. Surprisingly, the extract greatly enhanced cellular amyloid-beta peptide (Aβ) 42 productions in APP_NL_-H4 cells. Administration of leaf powder increased Aβ42 levels after 3 months and decreased levels after 12 months compared to control mice. Leaf powder had no effect on working memory after 3 months, but improved working memory after 12 months. Administration of UA decreased Aβ42 and P-tau levels and improved working memory after 12 months, similar to the administration of leave powder for 12 months. Amygdalin enhanced cellular Aβ42 production in APP_NL_-H4 cells, which was the same as the extract. Three-month administration of amygdalin increased Aβ42 levels slightly but did not significantly increase them, which is similar to the trend observed with the administration of leaf powder for 3 months. UA was likely the main compound contained in loquat leaves responsible for the decrease in intracerebral Aβ42 and P-tau levels. Also, amygdalin might be one of the compounds responsible for the transiently increased intracerebral Aβ42 levels.

## Introduction

Alzheimer’s disease (AD) is one of the most common neurodegenerative diseases. AD accounts for almost three-quarters of dementia cases, with the remainder accounted for by vascular dementia (VaD), mixed Alzheimer’s and VaD, dementia with Lewy bodies, and frontotemporal dementia^1^. The pathological proteins of AD mainly consist of amyloid-beta (Aβ) and phosphorylated tau (P-tau). Their deposition leads to neuronal damage through a series of pathways, which then induces memory and cognitive impairments^1,2^. However, clinical trials for AD that target the buildup of Aβ have been an ongoing disappointment. Several recent studies have reported the efficiency of anti-Aβ therapy. In 2021, the U.S. Food and Drug Administration approved aducanumab, an anti-Aβ antibody, for the treatment of AD. Moreover, another anti-Aβ antibody, lecanemab, showed positive results for AD treatment in 2022. The efficiency of anti-Aβ therapy remains controversial, but it is still important to search for a way to decrease Aβ deposition. Aβ is produced from amyloid precursor protein (APP) via sequential cleavage^3^. Since Aβ42 (42 amino acids), the most common species of Aβ, has a higher tendency to aggregate and form neurotoxic oligomers, Aβ deposition is closely related to an increase in Aβ42 levels^4^.

The loquat (*Eriobotrya japonica*) is a flowering plant with high medicinal value. Many parts of the loquat, such as the leaves, flowers, and kernels, have been used for the treatment of cough, inflammation, diabetes, cancer, pain, and other health issues^5^. In particular, loquat leaves have great potential for the treatment of inflammation, high fevers, chronic respiratory diseases, and gastroenteric disorders^6^. In addition, loquat leaves are used as an ingredient in tasty teas, known as ‘‘Biwa cha’’ in Japanese. Loquat leaves contain many bioactive components, such as triterpenoids and glycosides^7,8^. Ursolic acid (UA) is the predominant triterpenoid found in loquat leaves^9,10^ and exerts neuroprotective effects in several neurodegenerative animal models, such as Multiple sclerosis^11^, Parkinson disease^12^, depression^13^, and traumatic brain injury^14^. Amygdalin is a natural cyanogenic glycoside and is contained in loquat leaves, as well as kernels^8^. Amygdalin have been demonstrated to exert anti-oxidant^15,16^ and anti-cancer effects against several types of cancer^17,18^.

In this study, we investigated the effects of loquat leaf methanol extracts on Aβ42 production in human neuroglioma H4 cells stably expressing Swedish-type APP695 (APP_NL_-H4 cells). In addition, we administered loquat leaf powder to C57BL/6 J mice for 12 months and evaluated Aβ42 and P-tau levels, and working memory.

## Results

**Figure 1:**
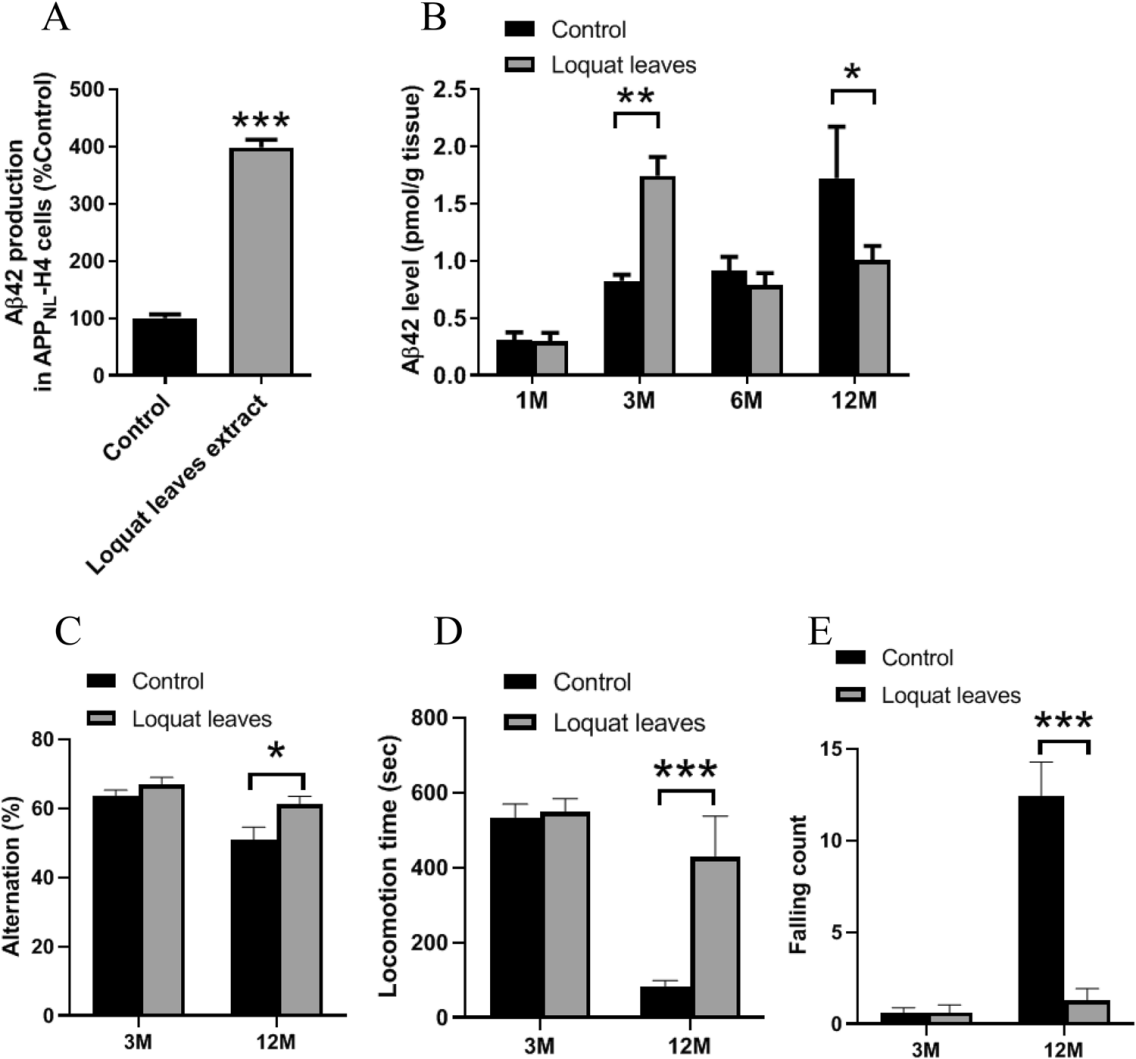
Effects of loquat leaves on Aβ42 level and mice behavior. Aβ42 production in APP_NL_-H4 cells treated with loquat leaf methanol extract for 48 h (**A**). Mice fed a powdered diet containing 5% loquat leaves for 1, 3, 6, and 12 months. Aβ42 levels in the mice cortex (**B**). Data are presented as the mean ± SEM, n = 6 per group. Working memory (Alternation) (**C**) in Y-maze test. Motor performance on the rotarod. Mice were assessed for locomotion time (**D**) and number of falls (**E**) during a period of 600 s. Data are presented as the mean ± SEM, n = 10 per group. Statistical analysis was performed using a student’s t-test (*p < 0.05; **p < 0.01; ***p < 0.001 vs. Control).

To investigate the effect of loquat leaf methanol extracts on Aβ42 production, we determined Aβ42 production in APP_NL_-H4 cells using ELISA. The extract increased Aβ42 production by approximately fourfold compared with that of the control (Fig. 1A). To investigate the effect of long-term dietary administration of powdered loquat leaves on intracerebral Aβ42 level in mice, we measured Aβ42 levels in the cortex by ELISA analysis. Mice administered loquat leaves showed increased Aβ42 levels after 3 months (Fig. 1B). The intracerebral Aβ42 levels increased in the control mice with aging. Loquat leaf powder administered mice showed decreased Aβ42 levels after 12 months compared to control mice (Fig. 1B). To evaluate whether Aβ42 levels affected working memory, we performed Y-maze tests. Mice administered loquat leaves exhibited the same level of alternation after 3 months and significant increase of alternation after 12 months in Y-maze task (Fig. 1C). Loquat leaves administered mice showed same level of locomotion time and falling count after 3 months, and higher locomotion activity and decreased falling count after 12 months in the rotarod test (Fig. 1D,E).

**Figure 2:**
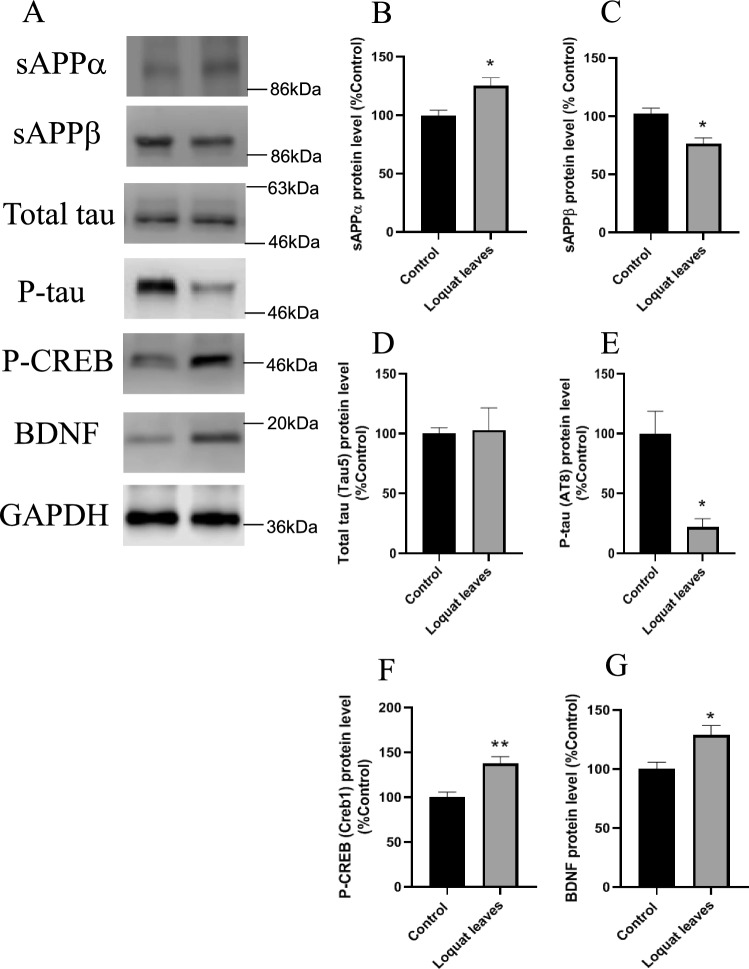
Effects of loquat leaves on APP cleavage, tau phosphorylation, and CREB-BDNF pathway. Mice fed a powdered diet containing 5% loquat leaves for 12 months. The protein level was determined by western blot analysis (**A**). Protein levels of sAPPα (**B**), sAPPβ (**C**), Total tau (**D**), P-tau (**E**), P-CREB (**F**), and BDNF (**G**) in the hippocampus. Data are presented as the mean ± SEM, *n* = 5 per group. Statistical analysis was performed using a student’s *t*-test (*p < 0.05; **p < 0.01 vs. Control).

We investigated the product levels of APP to elucidate the mechanism of decreased Aβ42 levels by loquat leaf powder after 12 months. The protein levels in loquat leaf powder administered mice showed increased sAPPα and reduced sAPPβ production (Fig. 2A–C). We evaluated the level of total tau and P-tau using Tau5 and AT8 antibodies, respectively (Fig. 2A,D,E). Administration of loquat leaves did not affect total tau levels (Fig. 2A,D) and decreased P-tau levels (Fig. 2A,E) compared to those in control mice. Administration of loquat leaves also increased phosphorylated CREB (P-CREB) and BDNF protein levels (Fig. 2A,F,G). We also evaluated these protein levels in loquat leaves administered to mice after 3 months. The 3-month administration of loquat leaves decreased the sAPPα protein level (Supplemental materials S2) and did not affect sAPPβ, P-tau, and P-CREB levels (Supplemental materials S2).

**Figure 3:**
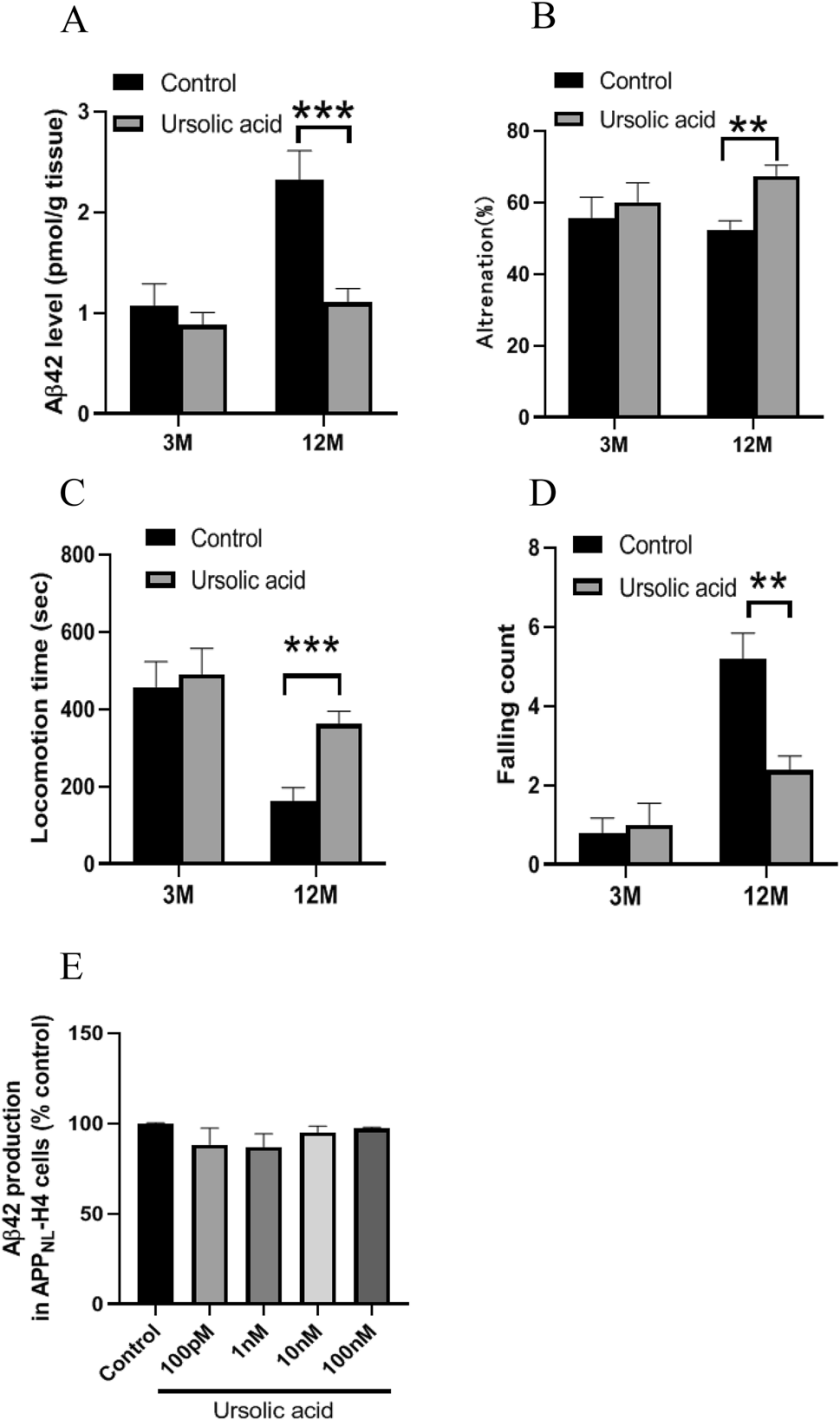
Aβ42 levels and mice behavior following administration of UA for 3 and 12 months. Mice fed with powdered diet containing 0.3125% UA for 3 and 12 months. Aβ42 production in the mice cortex (**A**). Data are presented as the mean ± SEM, n = 6 per group. Working memory (Alternation) (**B**) in Y-maze test. Motor performance on the rotarod. Mice were assessed for locomotion time (**C**) and number of falls (**D**) during a period of 600 s. Data are presented as the mean ± SEM, n = 10 per group. Statistical analysis was performed using a student’s *t*-test (**p < 0.01; ***p < 0.001 vs. Control). Aβ42 production in the APP_NL_-H4 cells treated with UA for 48 h (**E**). Data are presented as the mean ± SEM, n = 5 per group. Statistical analysis was performed using one-way ANOVA followed by post-hoc Newman-Keuls test.

Since UA is the predominant triterpenoid present in loquat leaves^9,10^, we investigated the effect of UA on Aβ42 production and behavior tests. UA administered mice showed the same levels of Aβ42 production after 3 months and decreased Aβ42 levels compared to control mice after 12 months (Fig. 3A). Although UA administration did not affect the behavior of mice after 3 months, UA increased alternation and locomotion time and decreased the falling count after 12 months (Fig. 3B–D). UA did not affect Aβ42 production in APP_NL_-H4 cells (Fig. 3E).

**Figure 4:**
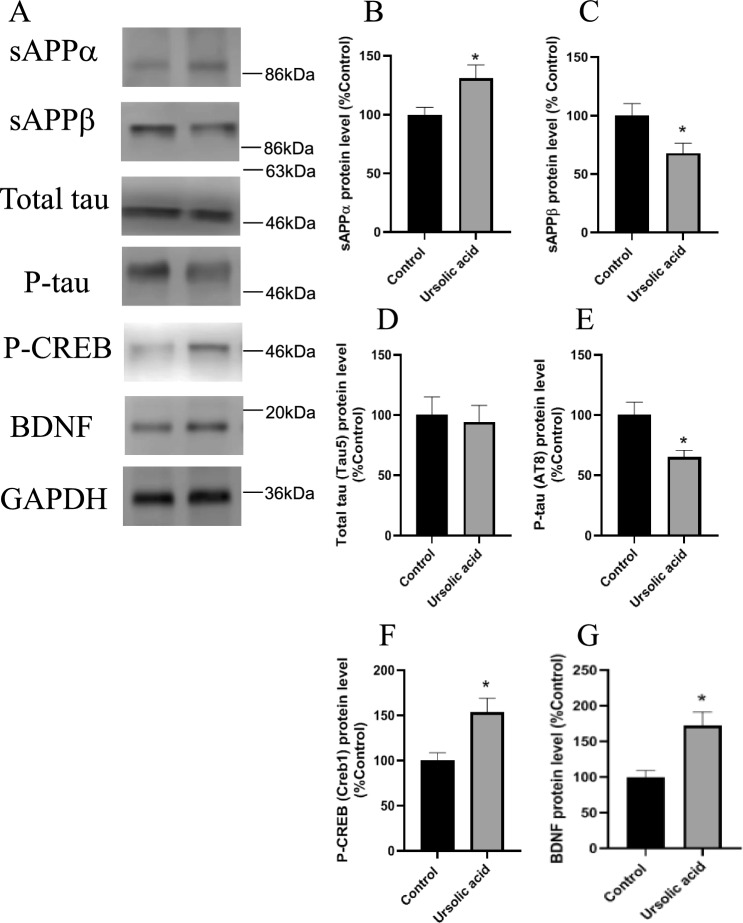
Effects of UA on APP cleavage, tau phosphorylation, and CREB-BDNF pathway. Mice fed a powdered diet containing 0.3125% loquat leaves for 12 months. The protein level determined by western blot analysis (**A**). Protein levels of sAPPα (**B**), sAPPβ (**C**), Total tau (**D**), P-tau (**E**), P-CREB (**F**), and BDNF (**G**) in the hippocampus. Data are presented as the mean ± SEM, *n* = 5 per group. Statistical analysis was performed using a student’s *t*-test (*p < 0.05 vs. Control).

Long-term administration of UA resulted in the same protein expression profile as loquat leaf powder administered mice. UA administered mice showed increased sAPPα levels and reduced sAPPβ production (Fig. 4A–C). UA treatment decreased the level of P-tau without altering total tau levels. (Fig. 4A,D,E). UA treatment increased P-CREB and BDNF protein levels (Fig. 4A,F,G).

**Figure 5:**
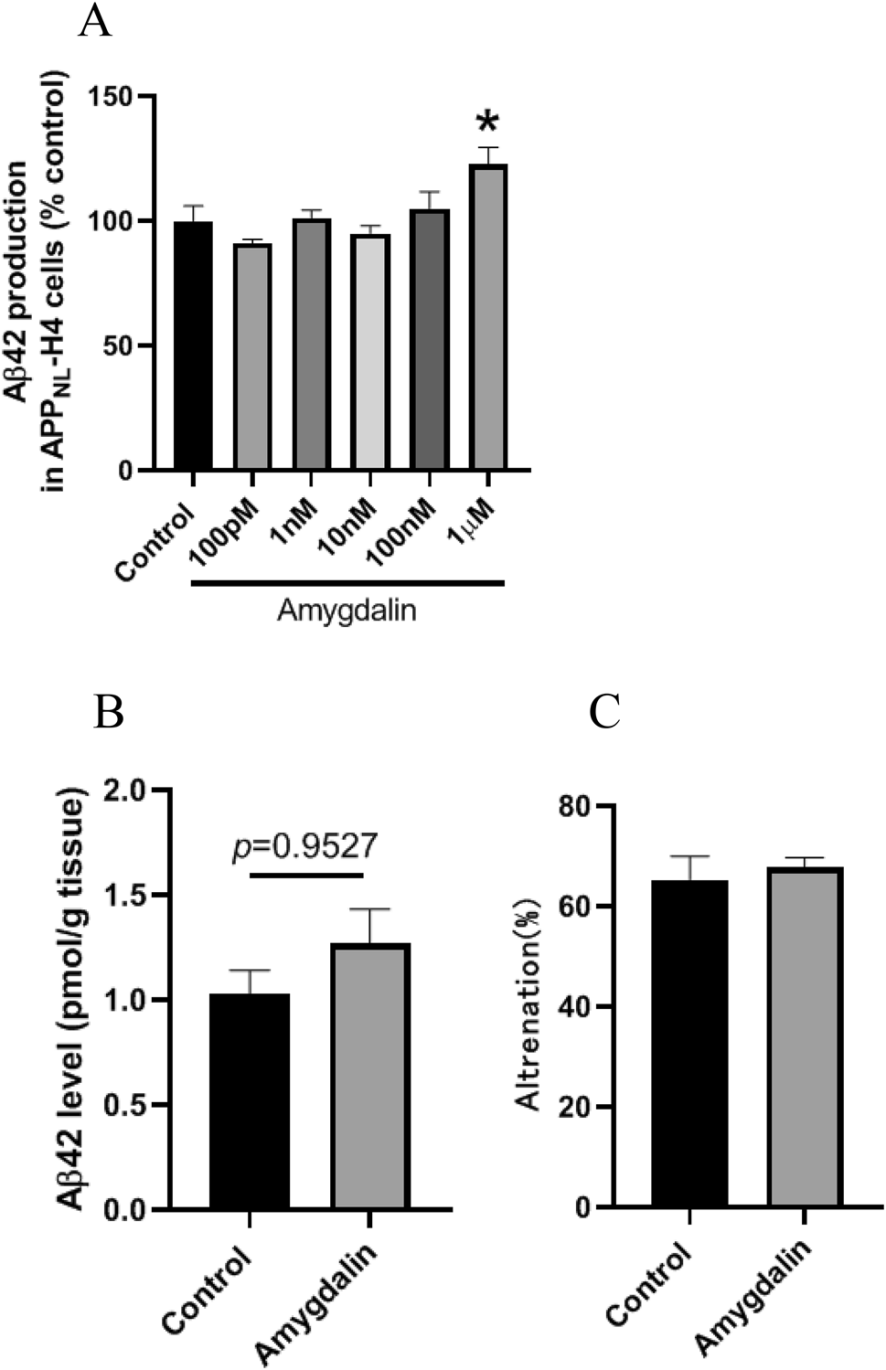
Effects of amygdalin on Aβ42 level and mice behavior. Aβ42 production in APP_NL_-H4 cells treated with amygdalin for 48 h (**A**). Data are presented as the mean ± SEM, n = 5 per group. Statistical analysis was performed using one-way ANOVA followed by post-hoc Newman-Keuls test (*p < 0.001 vs. Control). Mice fed a powdered diet containing 0.0695% amygdalin for 3 months. Aβ42 levels in the mice cortex (**B**). Working memory (Alternation) (**C**) in Y-maze test. Data are presented as the mean ± SEM, *n* = 5 per group. Statistical analysis was performed using a student’s *t*-test.

Amygdalin is also a biologically active compound contained in loquat leaves^8^. To investigate the effect of amygdalin on Aβ42 production, we examined cellular Aβ42 production. We found that 1 μM of amygdalin increased Aβ42 production in APP_NL_-H4 cells (Fig. 5A). In addition, mice fed with amygdalin showed slightly, but not significantly, increased Aβ42 levels after 3 months (Fig. 5B). No statistically significant differences were observed in alternation in the Y-maze test (Fig. 5C).

## Discussion

In this study, we found that a methanol extract of loquat leaves increased Aβ42 production in the APP_NL_-H4 cells. Mice fed loquat leaf powder after 3 months transiently increased intracerebral Aβ42 production, but decreased it after 12 months. Mice fed with loquat leaves for 12 months showed higher alternation task and motor function, which may be due to decreased Aβ42 and P-tau levels, and increased P-CREB and BDNF protein levels. We hypothesized that the major components of loquat leaves, UA and amygdalin, could influence Aβ42 production. Mice fed UA for 12 months exhibited the same results as mice fed loquat leaves for 12 months. These results suggest that UA was the main compound contained in loquat leaves responsible for decreased Aβ42 and P-tau levels and increased P-CREB, BDNF, alternation task, and motor function. In contrast, amygdalin increased cellular Aβ42 production in APP_NL_-H4 cells, with an increasing trend after 3 months of administration. It is possible that amygdalin is a factor contained in loquat leaves that increases cellular and intracerebral Aβ42 production.

The effects of loquat leaves and UA in Aβ peptide-induced AD mouse model have been reported in some studies. Loquat leaf extract^19^ and UA^20–22^ attenuated cognitive deficits induced by intracerebroventricular (i.c.v.) injection of the Aβ peptide. Thus, the neuroprotective effects of loquat leaves and UA on the impairment induced by exogenous Aβ peptides have been reported previously. However, the effects of loquat leaves and UA on natural brain aging accumulation of endogenous Aβ peptides and their long-term administration remain unclear. In this study, we first demonstrated that 12 months administration of loquat leaves and UA decreased intracerebral Aβ42 level, and increased working memory and motor function.

APP is cleaved into sAPPα and sAPPβ by α-secretase and β-secretase, respectively. Furthermore, APP C-terminal fragment produced by β-secretase is cleaved into Aβ by γ-secretase^23^. As the α-cleavage position is located in the middle portion of Aβ, this is called the non-amyloidogenic pathway. To understand the balance between the amyloidogenic and non-amyloidogenic pathways, we investigated the levels of sAPPα and sAPPβ. Administration of loquat leaves and UA increased sAPPα and decreased sAPPβ. These results suggest that loquat leaves and UA have the potential to shift APP processing to a non-amyloidogenic pathway, resulting in a decrease in intracerebral Aβ42.

Tau is a microtubule-associated protein containing many phosphorylated sites. Several studies have reported that environmental stress leads to an increase in P-tau^24–26^. We have also reported that intermittent hypoxia treatment causes an increase in P-tau via biological processes common to aging^27^. Moreover, it has been suggested that tau protein is a critical mediator of neuronal dysfunction and the associated cognitive and affective impairments seen after the experience of chronic stress^28^. In this study, we found that loquat leaves and UA decreased tau phosphorylation. The precise mechanisms are unknown, but loquat leaves and UA could suppress the effects caused by “environmental stress”.

We found that loquat leaves and UA enhanced the P-CREB and BDNF protein levels. CREB and BDNF are important molecules in learning and memory^29,30^. P-CREB induces the transcription of BDNF gene expression, which enhances synaptic activity in hippocampal neurons^31,32^. Aβ42 toxicity reduces learning and memory via downregulation of CREB phosphorylation and BDNF expression^33,34^. Mice fed loquat leaves and UA for 12 months showed higher working memory, which is due to the enhancement of P-CREB and BDNF by suppressing Aβ42 production.

We found that loquat leaf methanol extract and amygdalin increased cellular Aβ42 production in APP_NL_-H4 cells. Three months administration of loquat leaf powder increased intracerebral Aβ42 production transiently. In addition, amygdalin administration slightly, but not significantly, increased intracerebral Aβ42 levels after 3 months. Taken together, amygdalin might be one of the contributing factors contained in loquat leaves, which caused the increase in intracerebral Aβ42 levels after 3 months. Besides amygdalin, we speculated that another factor contributing to the increase in intracerebral Aβ42 levels could be the increase in γ-secretase by UA. *APH-1b*, a γ-secretase subunit, has a transcription factor binding site of p53 in the gene promotor (*APH-1b*, GeneCards database). The p53 signaling pathway is activated by UA^35,36^. Thus, γ-secretase activity induced by UA may increase Aβ42 levels after 3 months. For the decreased Aβ42 levels by UA after 12 months, UA shifts APP processing to a non-amyloidogenic pathway as mentioned above. Although loquat leaves elevated Aβ42 levels transiently, they did not exhibit disadvantages in working memory in the Y-maze test after 3 months. Previous research found that loquat leaf extract ameliorated cognitive deficits via i.c.v. injection of Aβ1–42 peptide^19^. However, we did not find a cognitive decline, even when Aβ42 levels were elevated. Therefore, the transient elevation of Aβ42 levels by loquat leaves was considered to have a low adverse effect.

In the current study, we demonstrated that long-term administration of loquat leaves and UA decreased Aβ42 and P-tau levels, which are indicators of neuronal toxicity and stress, respectively. Moreover, a decline in Aβ42 led to the maintenance of neuronal homeostasis to enhance working memory via the upregulation of CREB-BDNF signaling. Our data suggest that loquat leaves and the UA contained in these leaves have therapeutic implications in preventing the cumulative risk of AD.

## Methods

### Materials

All the plant experiments were in compliance with relevant institutional, national, and international guidelines and legislation. Loquat leaves were collected from Nagasaki prefecture in Japan. The samples collection was granted by Regulations for Handling Plant Collection for Research in Kyushu University (Fukuoka, Japan). The formal identification of loquat leaves performed by Dr. Shimizu (Kyushu University) and voucher specimens (No. EJ001) has been deposited in the Laboratory of Systematic Forest and Forest Products Sciences (Kyushu University)^9^. All leaves were air-dried and mechanically powdered. The methanol extracts of loquat leaves extracted with three times at room temperature^9^. UA (95%, 3-β-hydroxy-12-ursen-28-oic acid, CAS# 77–52-1) was purchased from Accela Chem Bio Co., Ltd (San Diego, CA). Amygdalin (95%, D-mandelonitrile 6-O-β-d-glucosido-β-d-glucoside, CAS# 29883-15-6) was purchased from Tokyo Kasei Kogyo Co. (Tokyo, Japan).

The following antibodies were used: anti-mouse/rat APP (597) (28,055, IBL, Gunma, Japan), anti- human sAPPβ-Wild Type (#18957, IBL), anti-Tau5 (ab82579, Abcam, Cambridge, MA), anti-AT8 (ab75603, Abcam), anti-cAMP response element binding protein 1 (CREB1, MAB18871, Abnova, Walnut, CA), and anti-brain derived neurotrophic factor (BDNF, bs-4989R, Funakoshi, Tokyo, Japan).

### Cell culture

H4 neuroglioma cells (APP_NL_-H4 cells) (Invitrogen Japan K.K., Tokyo, Japan) stably expressing Swedish-type mutated human APP695 were obtained^37^. APP_NL_-H4 cells exhibited enhanced Aβ production by increasing β cleavage of APP^38^. The cells were maintained in Dulbecco’s modified Eagle’s medium (DMEM; high glucose, Nacalai Tesque, Kyoto, Japan) with 10% (v/v) FBS, 5% amino acids, 1% penicillin–streptomycin (Invitrogen, Carlsbad, CA), and hygromycin (150 μg/ml) in a humidified atmosphere with 5% CO_2_ at 37 °C.

### Animal procedures

Male WT C57BL/6J mice (Tokyo Laboratory Animals Science, Tokyo, Japan) were housed in appropriate animal care facilities at Saitama Medical University (Saitama, Japan) and handled in accordance with established international guidelines. The experimental protocols were approved by the Animal Research Committee of Saitama Medical University. Mice were maintained on a 12 h/12 h light/dark cycle with free access to a powdered diet (CLEA Japan, Tokyo, Japan) and tap water. Five-week-old mice were fed a powdered diet containing 5% loquat leaf powder for 1, 3, 6, and 12 months. Previous reports demonstrated that UA and amygdalin contained 6.25% and 1.39% weight in dry weight of loquat leaves, respectively^8,9^. Based on these findings, the doses of UA and amygdalin were calculated to be equivalent to those contained in loquat leaves. The mice were fed a powered diet containing 0.3125% weight of UA for 3 and 12 months, and 0.0695% weight of amygdalin was administered for 3 months. The average daily food intake of mice was 4.6 g, including 230 mg/day of loquat leaf powder (5% weight), 14.4 mg/day of UA (0.3125% weight), and 3.2 mg/day of amygdalin (0.0695% weight). No difference in body weights were observed between control (26.1 ± 0.24 g) vs mice administered loquat leaf powder after 3 months (25.4 ± 0.27 g), control (31.1 ± 0.28 g) vs mice administered loquat leaf powder after 12 months (29.3 ± 0.80 g), control (26.1 ± 0.17 g) vs mice administered UA after 3 months (25.5 ± 0.26 g), control (31.2 ± 0.28 g) vs mice administered UA after 12 months (31.7 ± 0.88 g), and control (25.1 ± 0.30 g) vs mice administered amygdalin after 3 months (24.2 ± 0.32 g). All animal studies were approved by the Institutional Animal Care Committee of Saitama Medical University. Confirming that all experiments were performed in accordance with relevant guidelines and regulations.

### ELISA analysis

APP_NL_-H4 cells were plated at a density of 500,000 cells/well in 6-well plates. After resting for 24 h, the cells were treated with 1 ppm methanol extract of loquat leaves, UA or Amygdalin for 24 h, and the medium was replaced with the same medium containing the extract or amygdalin. The cells were incubated for 24 h and the medium was harvested. Aβ total of 42 levels in the medium from the APP_NL_-H4 cells were measured using ELISA kits (IBL, Ltd., Fujioka, Japan).

Mouse cortices were homogenized with a tissue homogenizer (Rotor: TLA45, Beckman Coulter) on ice in Tris-buffered saline (TBS) (25 mM Tris–HCl pH 7.4, 137 mM NaCl, 2.68 mM KCl) containing a protease inhibitor cocktail (Roche Diagnostics Ltd, Mannheim, Germany) and phosphatase inhibitor cocktail (Nacalai Tesque). The homogenates were ultracentrifuged at 45,000×*g* for 20 min at 4 °C. The cortical Aβ42 levels were determined using a Human/Rat β amyloid (42) ELISA Kit (Wako, Tokyo, Japan). The results were measured using a Benchmark Microplate Reader (Bio-Rad, Hercules, CA, USA).

### Western Blotting

Mouse hippocampi were homogenized on ice in Tris-buffered saline (TBS) containing a protease and phosphatase inhibitor cocktail. The homogenates were ultracentrifuged at 45000×*g* for 20 min at 4 °C. Protein concentrations were determined using the BCA protein assay kit (Nacalai Tesque, Tokyo, Japan). Proteins (10 μg/lane) in the lysates were subjected to SDS–polyacrylamide gel electrophoresis and transferred to nitrocellulose membranes (Bio-Rad). After blocking with 5% skim milk (MEGMILK SNOW BRAND Co., Ltd., Tokyo, Japan) in PBS containing 0.05% Tween 20 (Polyoxyethylene Sorbitan Monolaurate, Nacalai Tesque) (PBS-T), the membranes were incubated with the primary antibodies overnight, followed by incubation with horseradish peroxidase-conjugated secondary antibodies (Cell Signaling Technology, Beverly, MA) and washing with PBS-T three times. The membranes were then incubated with a chemiluminescence reagent (Chemi-Lumi One Super, Nacalai Tesque; ImmunoStar LD, FUJIFILM). Images of the membranes were captured using a C-DiGit blot scanner (LI-COR, Lincoln, NE, USA) and subjected to ImageJ analysis. Each membrane was probed with an anti-GAPDH antibody (1:1000, ABS16, Millipore, Billerica, MA, USA), and the bands were used as loading controls. A prestained molecular weight marker was used to confirm the expected size of the target proteins. For some figures, unrelated lanes were cropped out. Full size images are provided in the Supplementary materials S1, S3, and S4.

### Behavioral tests

The Y-maze apparatus (Hazai-ya, Tokyo, Japan) was a 3-arm radial maze with equal angles between all arms (8 cm width, 40 cm length, and 15 cm height). Mice were tested individually by placing them in an arm of the maze and allowing them to move freely throughout the 3 different arms for 10 min. The sequence and entries into each arm were recorded. An alternation was determined from successive consecutive entries into the 3 different arms on overlapping triads in which all arms were represented. For example, ACBABCABAB, a sequence of entries to the 3 arms A, B, or C, would generate 5 ‘successful’ alternations, ACB, CBA, ABC, BCA, and CAB; the total number of possible alternations corresponded to the number of the total arm entries minus 2 (in this example, the total number would equal 8). The percentage alternation was calculated as (the number of ‘successful’ alternations divided by the number of the total arm entries minus 2) × 100^27,39^.

We used an accelerating rotarod treadmill for mice (Mouse Rotarod, UgoBasile, Italy) to evaluate motor function. All mice were tested on the rotarod at 28 rpm. The time each mouse stayed on the rod (locomotion time) was recorded by a trip switch under the floor of each rotating drum, with a maximum recording time of 600 s. If the mouse fell, it was placed back onto the cylinders for the remainder of the 600 s trial and the number of falls was counted (falling count)^40,41^.

### Statistical analyses

Two-sample comparisons were carried out using Student’s *t*-test. Multiple comparisons were performed by one-way ANOVA followed by Newman-Keuls post-hoc test. All data were analyzed using Graph Pad Prism ver.8.01 (Graph Pad Software, Pad Software, Inc., San Diego, CA), and expressed as mean ± SEM. *p* values < 0.05 were considered statistically significant (*p < 0.05, **p < 0.01, ***p < 0.001).

## Ethics approval

We confirm that all methods were performed in accordance with the relevant guidelines and regulations. The study was carried out in compliance with the ARRIVE guidelines (https://arriveguidelines.org).

### Supplementary Information

Supplementary Information.

## Data Availability

The datasets generated during and/ or analyzed during the current study are available from the corresponding author on request.
