# Supplementary material for: Long term administration of loquat leaves and their major component, ursolic acid, attenuated endogenous amyloid-β burden and memory impairment
Source: Sci Rep. 2023 Oct 5;13:16770. doi: 10.1038/s41598-023-44098-3 (PMC10556093; doi:10.1038/s41598-023-44098-3)
Supplement: Supplementary file 1 — Supplementary Information. [file 41598_2023_44098_MOESM1_ESM.pdf]

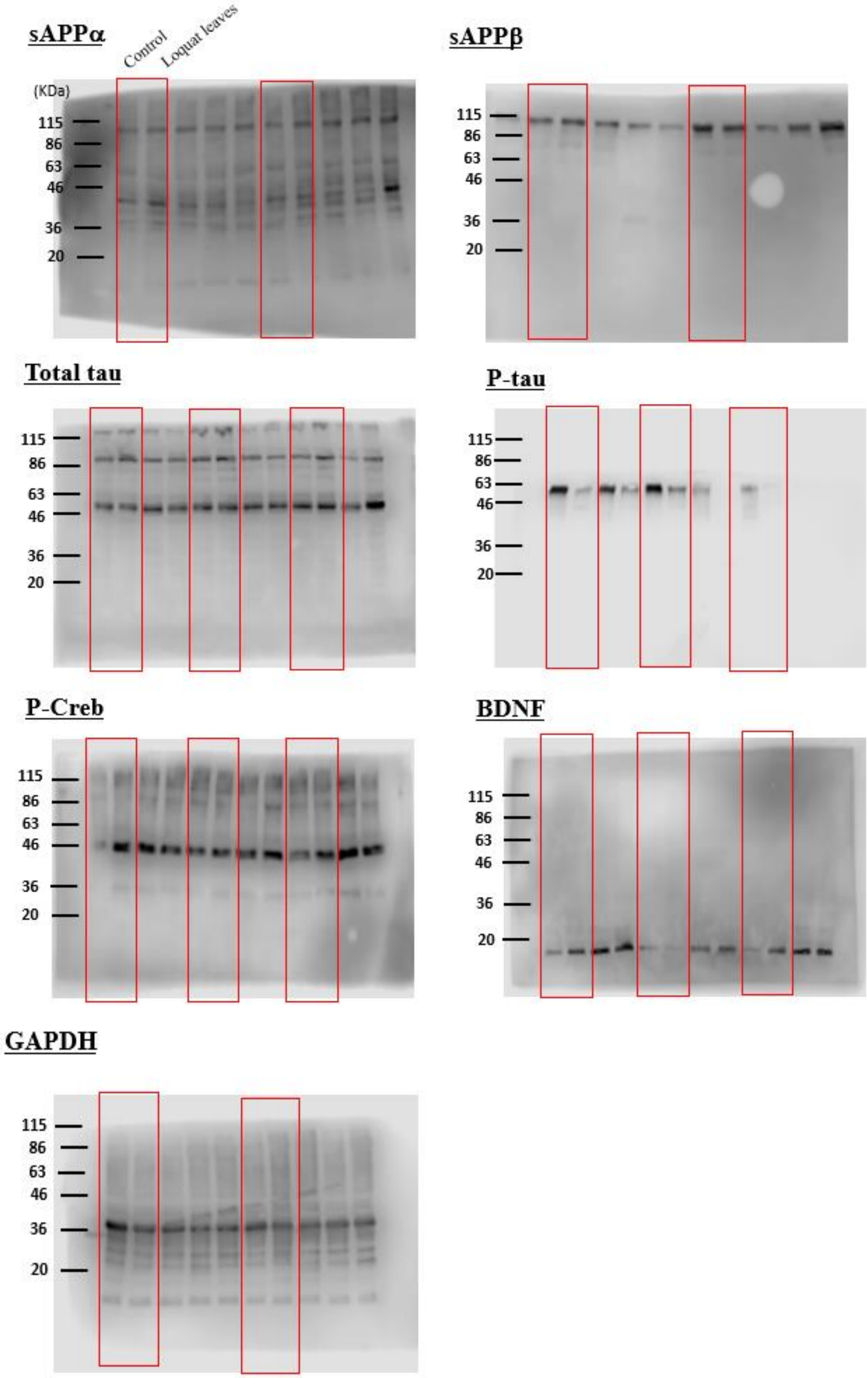

Supplemental materials S1

Full length of image of western blotting for Figure 2A.

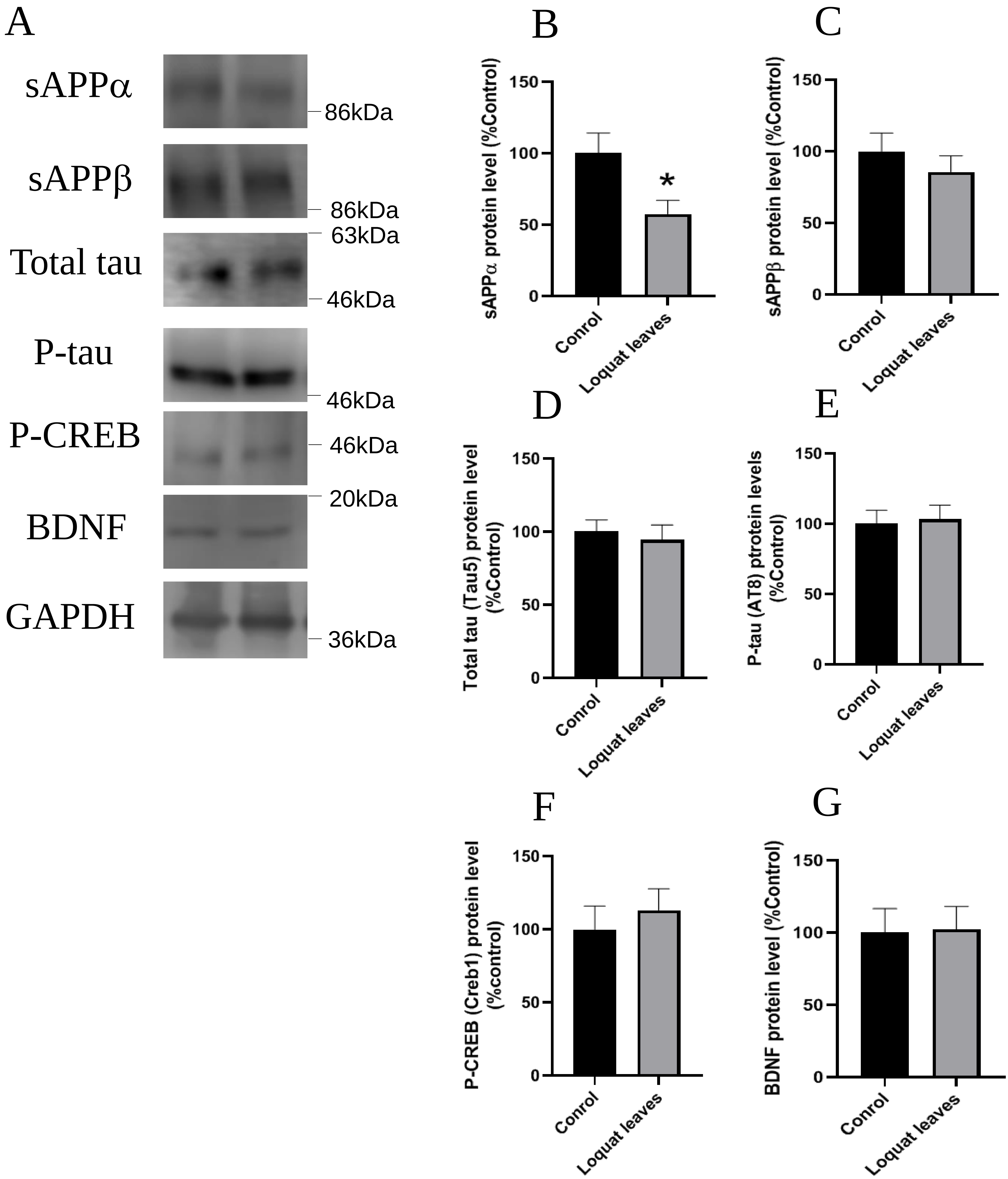

Supplemental materials S2

Effects of loquat leaves for 3 months on APP cleavage, tau phosphorylation, and CREB-BDNF pathway.

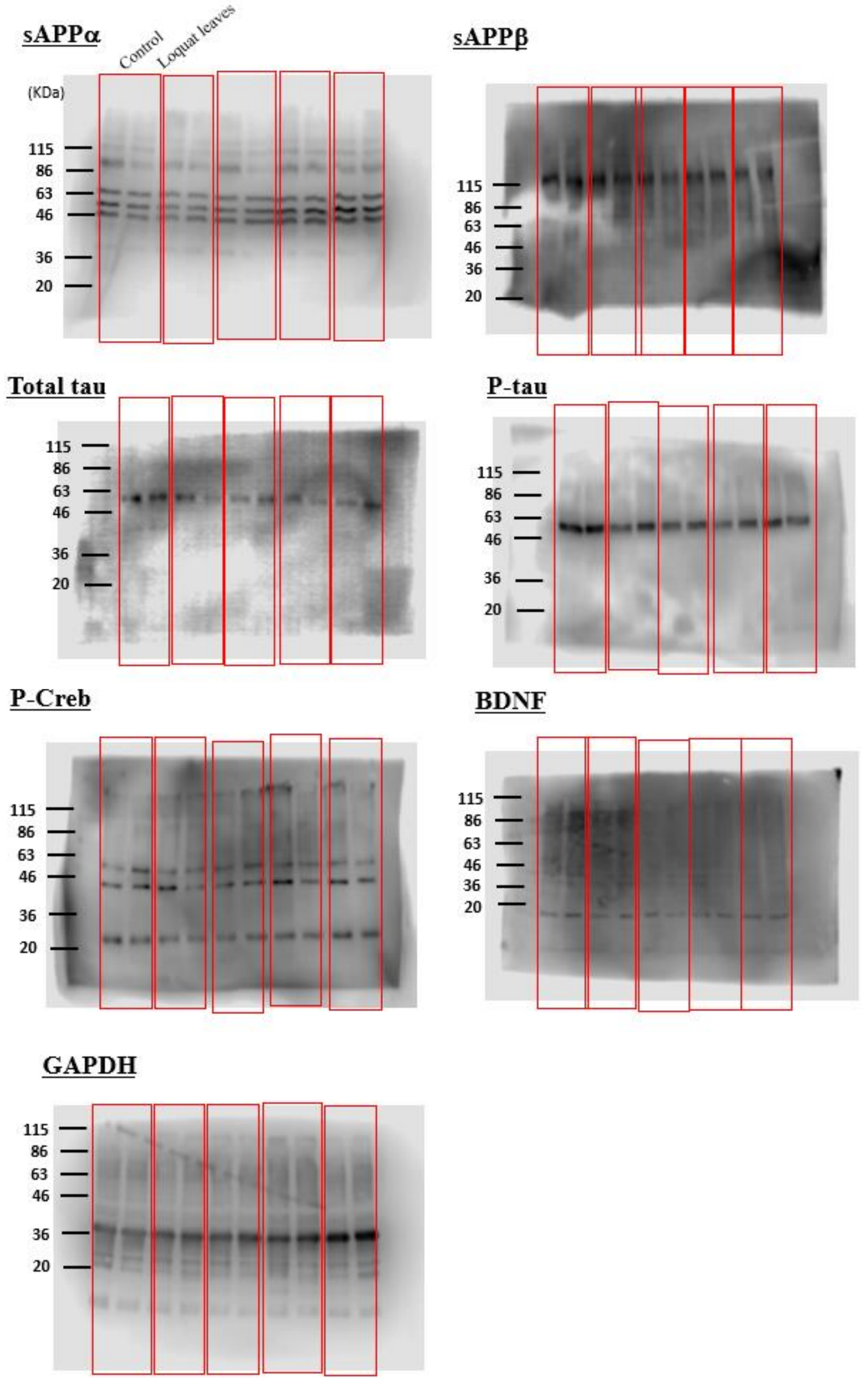

Supplemental materials S3

Full length of image of western blotting for Supplemental materials S2.

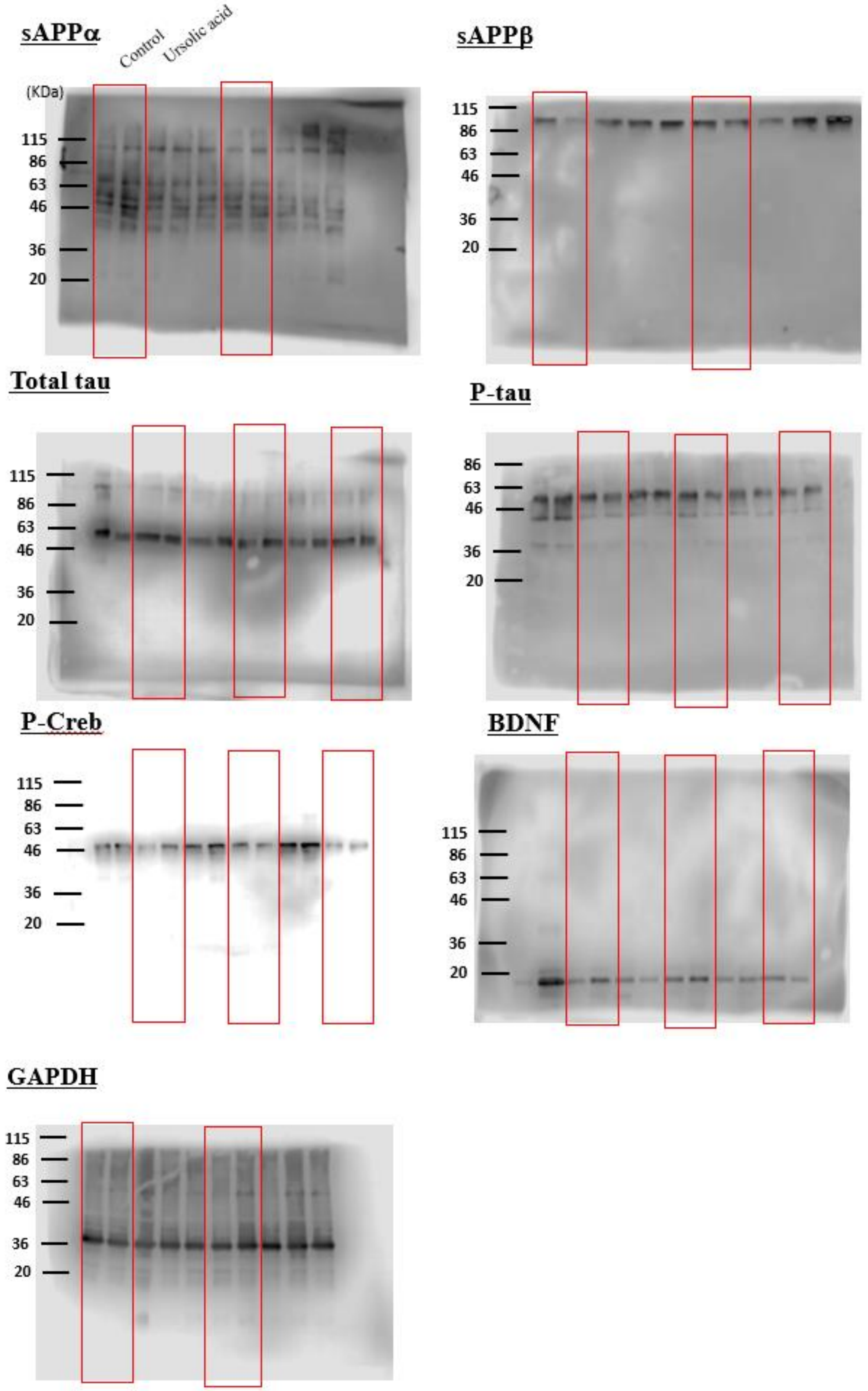

Supplemental materials S4

Full length of image of western blotting for Figure 4A.
